# Septins are critical regulators of osteoclastic bone resorption

**DOI:** 10.1038/s41598-018-31159-1

**Published:** 2018-08-29

**Authors:** Anaïs M. J. Møller, Ernst-Martin Füchtbauer, Annemarie Brüel, Thomas L. Andersen, Xenia G. Borggaard, Nathan J. Pavlos, Jesper S. Thomsen, Finn S. Pedersen, Jean-Marie Delaisse, Kent Søe

**Affiliations:** 1 Department of Clinical Cell Biology, Vejle Hospital/Lillebaelt Hospital, Institute of Regional Health Research, University of Southern Denmark, Beriderbakken 4, 7100 Vejle, Denmark; 2 Department of Molecular Biology and Genetics, Aarhus University, 8000 Aarhus, Denmark; 3 Department of Biomedicine, Aarhus University, 8000 Aarhus, Denmark; 4 Cellular Orthopaedic Laboratory, School of Biomedical Sciences, University of Western Australia, Nedlands, 6009 Australia

## Abstract

Septins are known to play key roles in supporting cytoskeletal stability, vesicular transport, endo-/exocytosis, stabilizing cellular membranes and forming diffusion barriers. Their function in mammalian cells is poorly investigated. The osteoclast offers an interesting tool to investigate septins because all cellular activities septins were reported to be involved in are critical for osteoclasts. However, the existence of septins in osteoclasts has not even been reported. Here we show that the *SEPT9* gene and Septin 9 (SEPT9) protein are expressed and synthesized during differentiation of human osteoclasts. Pharmacological stabilization of septin filaments dose dependently inhibits bone resorption of human osteoclasts *in vitro* suggesting a role for septins in bone resorption. Attesting to this, conditional deletion of *Sept9* in mice leads to elevated levels of trabecular bone and diminished femoral growth *in vivo*. Finally, systematic interrogation of the spatial organization of SEPT9 by confocal microscopy reveals that SEPT9 is closely associated to the structures known to be critical for osteoclast activity. We propose that septins in general and SEPT9 in particular play a previously unappreciated role in osteoclastic bone resorption.

## Introduction

Septins are a family of filamentous proteins that are considered as the fourth component of the cytoskeleton, joining the other three well characterized cytoskeletal components: actin, microtubules and intermediate filaments^1^. First discovered in *Saccharomyces cerevisiae*^2^, septins are also expressed in multicellular organisms ranging from *Caenorhabditis elegans* to humans. Two septins were identified in *C*. *elegans*, while seven have been identified in *S*. *cerevisiae* and 13 in humans (SEPT1 to 12 and SEPT14)^3^. Septins were originally identified due to the explicit role of four genes (CDC3, 10, 11 and 12) in cytokinesis in budding yeast^2^, and were later given the name “septins” due to their clear appearance at the septa of budding yeast^4^.

Compared to lower organisms, the function of the 13 human septins is poorly understood. They are divided into subgroups based on sequence homology. The SEPT2-subgroup encompasses SEPT1, SEPT2, SEPT4 and SEPT5; SEPT3-subgroup: SEPT3, SEPT9 and SEPT12; SEPT6-subgroup: SEPT6, SEPT8, SEPT10, SEPT11 and SEPT14; SEPT7-subgroup: SEPT7^1^. Septins appear unstable as monomers but form spontaneous, heteromeric, palindromic filaments^5^. The minimal subunit of such filaments is composed of septins from subgroups 7:6:2:2:6:7^6^. However, in the presence of SEPT3 subgroup members, particularly SEPT9, octamers of the composition 9:7:6:2:2:6:7:9 are formed^7^. Depending on the local environment, salt concentration etc. they are also able to form filaments or rings^1^. Human septins have been found to function as lateral diffusion barriers at the cellular membrane of polarized epithelial cells^8,9^, at the primary cilium^10^, to be important for exocytosis/vesicular transport^9,11,12^, bacterial entry into host cells^13^, phagosomes^14^ etc. Finally, a recent study reported through genome-wide association studies that single nucleotide polymorphisms in SEPT5 may be associated with low bone mineral density^15^.

SEPT9 in particular is not well understood in the human setting, but it has been suggested to be a tumor suppressor as well as a proto-oncogene^16^ and mutations in the *SEPT9* gene have been shown to cause hereditary neuralgic amyotrophy^17^ due to loss of proper hetero-octamer formation with other septins^18^, disruption of proper microtubule bundling^19^ and impaired vesicular transport^11^. A role of SEPT9 in bone development and homeostasis is suggested by a number of observations: SEPT9 mediates the binding of hetero-octamers to actin^20,21^ and microtubules^7^, it is involved in vesicular transport^11^, it acts as a diffusion barrier at the base of membrane protrusions (such as cilia, filopodia and pseudopodia)^8,22^ and patients carrying somatic mutations in *SEPT9* have a short stature and craniofacial characteristics^23^. When considering the general implications of septins on the cytoskeleton, vesicular transport, exo- and endocytosis and on skeletal growth it is likely that a cell, which is essential for skeletal growth and is a strongly mobile and secretory cell, such as the osteoclast (OC), may be dependent on the action of septins. Therefore, we investigated if septins, in particular SEPT9, play an important role in the activity of OCs.

OCs are large multinucleated cells and their formation through differentiation and multiple fusions of monocytic precursors is a prerequisite for efficient degradation of bone. The OC resorbs bone within a sealing zone containing F-actin and podosomes. It does so by secreting protons onto the bone surface in order to dissolve the mineral. Furthermore, a cocktail of proteases (e.g. cathepsin K) is released into the resorptive lacunae in order to degrade the vast network of mostly collagen type I fibres^24^. When OCs resorb bone they either make a series of round resorption cavities (pits) where the OC is immobile during but mobile between periods of resorption or it resorbs bone while moving, making elongated resorption cavities (trenches)^25^. An OC resorbing bone requires an efficient coordination between exo-and endocytosis and it occurs with high fidelity^25^. This may require diffusion barriers at the level of the ruffled border to facilitate a separation of these two processes, but it also requires an efficient and fast transport along microtubules and actin filaments, a function often ascribed to septins. At present the role of septins in OC function is unknown. Among the septins expressed in mammalian cells, SEPT9 is interesting because it bridges the interaction between the cytoskeleton, membranes etc. to the septin octamers^7,16,17,19,21^. Thus, SEPT9 may serve as an archetype member to address the role of septins in osteoclastic resorption.

In the present study, we document the existence of SEPT9 in human OCs. By Q-RT-PCR and Western blotting we show that SEPT9 is differentially expressed as splice variants during OC differentiation. In addition, we detail the subcellular localization of SEPT9 with respect to the cytoskeleton and endocytic markers in bone resorbing OCs by confocal microscopy. Moreover, we demonstrate that pharmacological blockade of septins by forchlorfenuron (FCF), a drug which prevents the turnover of septin filaments, attenuates bone resorption with selective inhibition to those OCs making trenches compared to those forming isolated pits. Consistently, we provide evidence that conditional ablation of the *Sept9* gene in mice leads to elevated levels of trabecular bone and diminished femoral growth *in vivo*.

## Results

### The *SEPT9* gene is expressed during human OC differentiation

**Figure 1:**
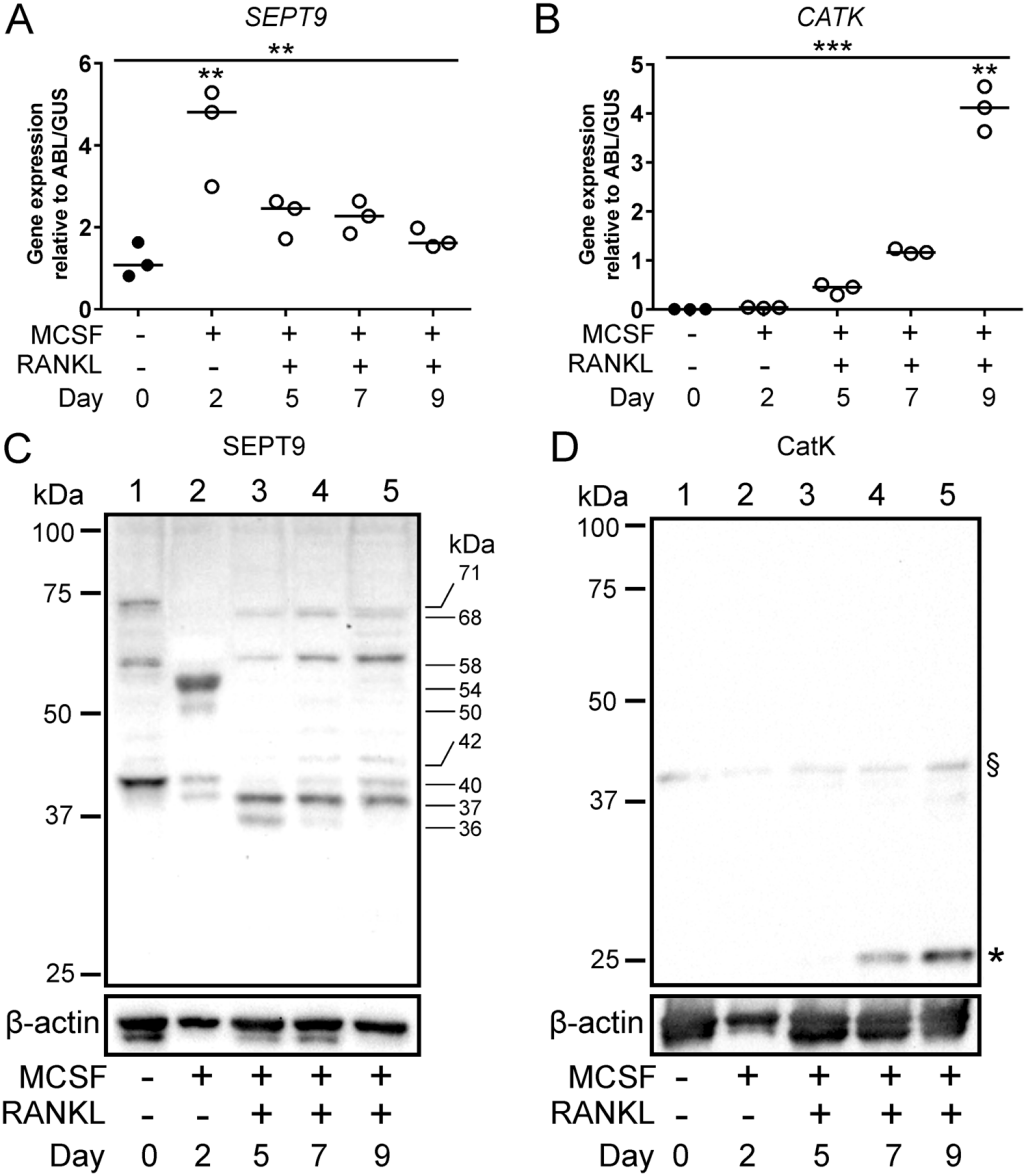
The *SEPT9* gene is expressed during human OC differentiation and different isoforms of SEPT9 protein can be detected. (**A**) Q-RT-PCR of *SEPT9*, n = 3 biological replicates, representative of three independent experiments with similar results. Statistics: Kruskal-Wallis test, two-tailed, **p = 0.0015; Dunn’s multiple comparisons test, **p = 0.0076. (**B**) Q-RT-PCR of *CATK*, n = 3 biological replicates (same as in A), representative of three independent experiments with similar results. Statistics: Kruskal-Wallis test, two-tailed, ***p < 0.0001; Dunn’s multiple comparisons test, **p = 0.0041. (**C**) Western blot of SEPT9 protein representative of three independent experiments with similar results. (**D**) Western blot of CatK protein (same cell lysates as in C representative of three independent experiments with similar results. §, pro-CatK; *, active CatK. C and D show cropped Western blot images; full size uncropped unedited blots can be seen in Supplementary Figure S1.

We prepared cell lysates and followed gene expression of *SEPT9* during differentiation of human CD14^+^ monocytes into mature OCs. Figure 1A shows that CD14^+^ monocytes express the *SEPT9* gene and that the expression level is significantly induced about 5-fold after a 2-day exposure to macrophage colony-stimulating factor (MCSF). The expression level drops by about 50% after 3 additional days of exposure to receptor activator of nuclear factor kappa-B ligand (RANKL) and reaches a stable level for the remainder of the differentiation. The PCR probe spans two exons, which are present in all known splice variants of *SEPT9*. Thus, this probe is able to detect the expression of all the known splice variants. The expression pattern of SEPT9 is very different from the RANKL-induced expression of the *CATK* gene (Fig. 1B).

### Different splice variants of SEPT9 are expressed during OC differentiation

Western blotting analyses of cell lysates collected during OC differentiation clearly reveal that SEPT9 protein is detected at all stages of differentiation, but also that possible isoforms are dominating depending on the differentiation stage (Fig. 1C). It is well documented that the human SEPT9 gene can be expressed as up to 18 splice variants resulting in SEPT9 protein with predicted MWs ranging from roughly 38 to 69 kDa^26^ and that their expression varies among different tissues and pathologies^26–30^. It is therefore relevant to address which splice variants may be synthesized into SEPT9 protein in OCs. In the CD14^+^ monocytes at day 0 three different isoforms are expressed. The variant running with an approximate MW of 40 kDa is clearly dominating, but also other variants with apparent MWs of 58 and 71 kDa are detected. On day two of MCSF exposure *SEPT9* mRNA expression peaks. A variant running at 54 kDa is most abundant, while only minor bands are visible at 50, 40 and 37 kDa. Once differentiation towards matured OCs is initiated by adding RANKL, the variant at 54 kDa is completely lost. Instead, there is over time a steady expression of a variant running at 37 kDa and an increasing presence of variants running at 58 and 68 kDa. The variant running at 36 kDa is only clearly detected on day 5, while weak bands at 40 and 42 kDa only become visible on days 7 and 9. These data show that there is a dynamic regulation of the SEPT9 variants in monocytes, macrophages and OCs. In addition, quantifications of the Western blots show that the overall protein level of SEPT9 (data not shown) reflects well the mRNA expression levels. This unchanged level of SEPT9 protein synthesis is in contrast to a classical OC protein, cathepsin K, since the synthesis of this protein is clearly enhanced upon addition of RANKL (Fig. 1D).

### Forchlorfenuron, a stabilizer of septin filaments, inhibits osteoclastic bone resorption and in particular OCs making trenches

**Figure 2:**
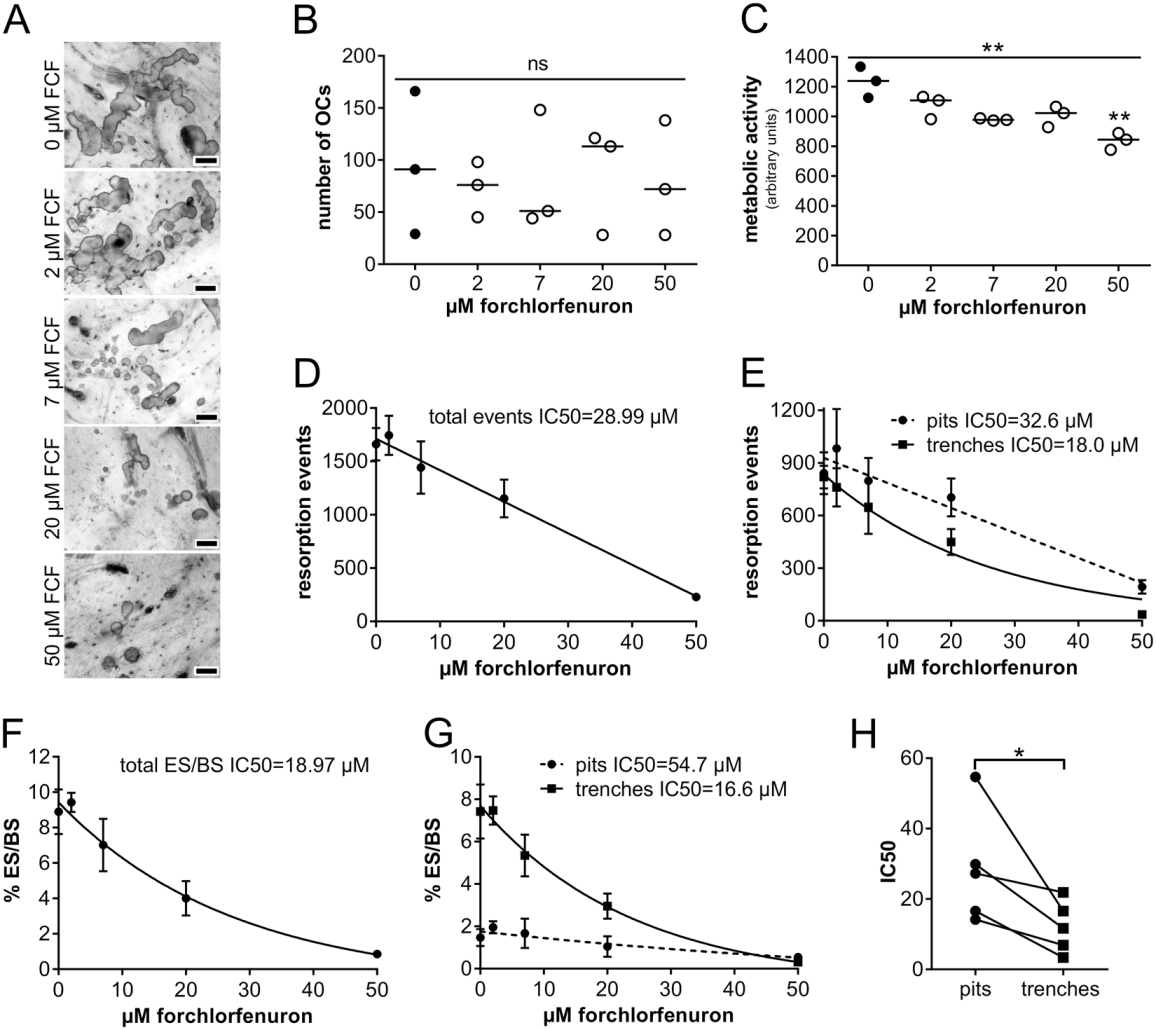
FCF, which stabilizes septin fibers, inhibits human OC resorption and in particular OCs making trenches. (**A**) Bone slices stained with toluidine after exposing bone resorbing OCs to various concentrations of FCF. Scale bar: 50 µm. (**B**) Total number of TRAcP^+^ cells with 2 nuclei or more per bone slice with and without FCF. Statistics: Kruskal-Wallis test, two-tailed, p = ns (not significant); horizontal line indicates the median. (**C**) Metabolic activity of cells seeded on the same bone slices as shown in A. Statistics: Kruskal-Wallis test, two-tailed, **p = 0.0014; Dunn’s multiple comparisons test, **p = 0.0056 compared to 0 µM FCF; horizontal line indicates the median. (**D**) IC50 of FCF with respect to the total number of resorption events per bone slice. Linear fit, r^2^ = 0.92; IC50 = 28.99 µM. (**E**) IC50 of FCF based on data shown in D, but sub-split into pits and trenches. Pits: linear fit, r^2^ = 0.79. IC50 = 32.6 µM; Trenches: non-linear fit, r^2^ = 0.90. IC50 = 18.0 µM. (**F**) IC50 of FCF with respect to the total eroded surface (ES). Non-linear fit, r^2^ = 0.92. IC50 = 18.97 µM. (**G**) IC50 of FCF based on data shown in F, but sub-split into pits and trenches. Pits: non-linear fit, r^2^ = 0.53. IC50 = 54.65 µM; Trenches: non-linear fit, r^2^ = 0.93. IC50 = 16.57 µM. (**D**–**G**) Each data point represents the mean ± SD (n = 4) from a representative experiment (a total of five independent experiments). (**H**) Comparison of FCF IC50s for pits and trenches, respectively, showing the means from five independent experiments. Statistics: Wilcoxon matched-pairs signed rank test, one-tailed, *p = 0.03.

Given that SEPT9 is expressed in OCs we next investigated whether SEPT9 influences OC function. Towards this we used FCF, a pharmacological stabilizer of septin filaments, to interrogate the potential contributions of septin during osteoclastic bone resorption^31–33^. Although FCF is a pan-inhibitor of all septins, SEPT9 forms the outer most septin in these octamer filaments and mediates the interaction to e.g. actin and microtubules and thus is most susceptible to the FCF inhibition^1,5,7,22,29,34^. In the literature, effective doses of FCF ranging from 5 µM to 2 mM have been employed on different cells in culture^31–33,35^. We experienced that FCF in some experiments was partly cytotoxic at 100 and strongly at 200 µM (as determined by metabolic activity, data not shown). Therefore, in our experiments we focused on doses ranging between 2 and 50 µM FCF. Figure 2A, D and F show that FCF inhibits bone resorption by human OCs in a dose dependent manner in the absence of cellular toxicity (Fig. 2B and C); although a slight reduction in metabolic activity was observed (Fig. 2C). However, it is also evident that the two resorption modes of the OC are not equally sensitive. Figure 2E and G show that the continuous resorption mode of the OC, resulting in trenches, is more sensitive than the OCs resorbing intermittently making pits. Figure 2H shows that this difference in sensitivity was reproduced in five independent experiments.

### SEPT9 shows distinct localizations at several sites in trench-forming OCs

**Figure 3:**
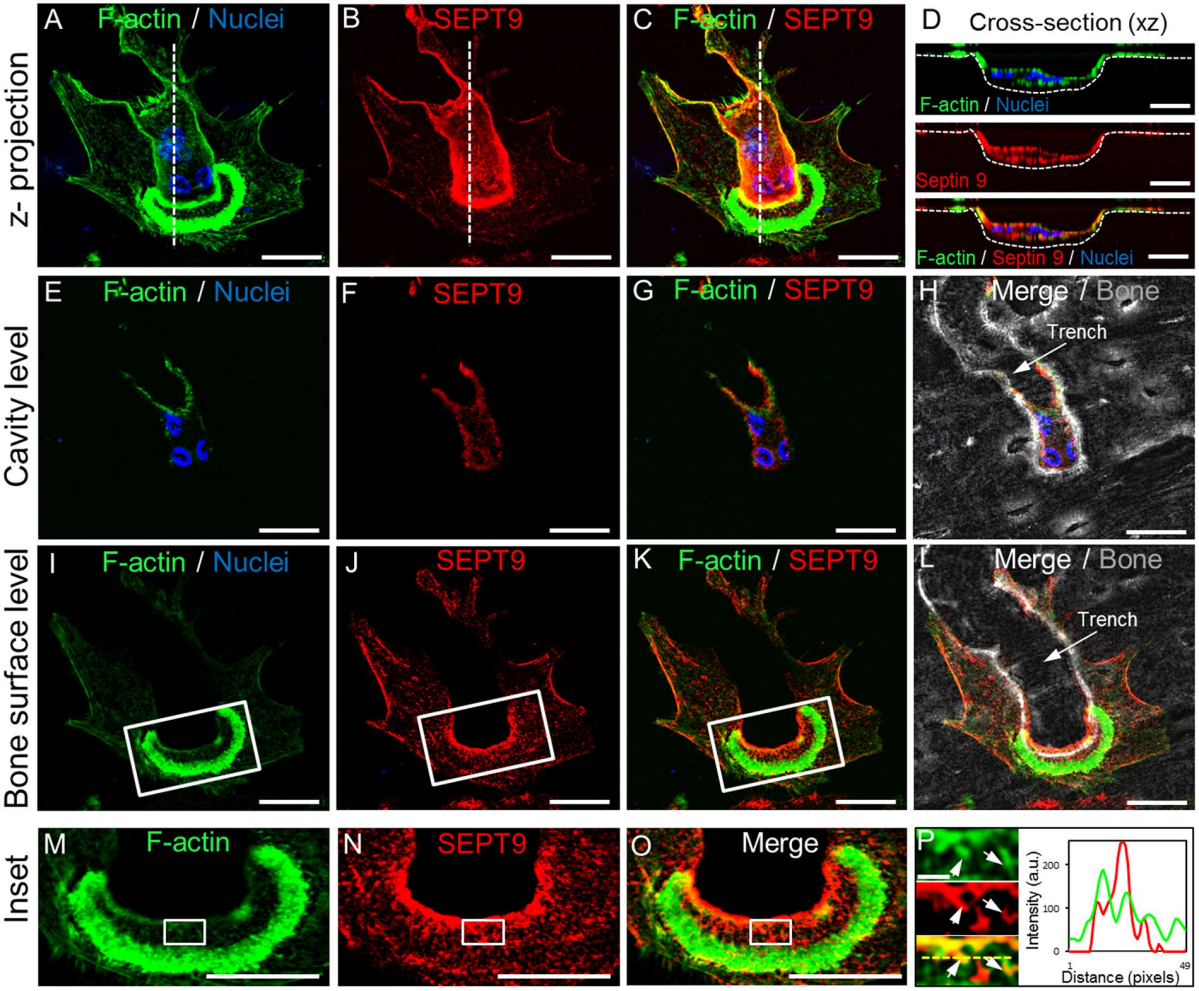
Confocal IF detection of SEPT9 shows that it is primarily allocated to the sites of the human OC engaged in active bone resorption and to those located within the boundaries of the trench. (**A**–**C**) 3D z-projection of trench resorbing OC. Scale bar: 20 µm. Please refer to Supplementary Video S1 for a rotating version of Fig. 3C. (**D**) Cross-section (xz) through the length axis of the OC and trench as indicated by white dashed lines in A to C. Scale bars: 20 µm. E-H) A single confocal plane (0.55 µm) (xy) at the trench level within the cavity. Scale bars: 20 µm. (**I**–**L**) A single confocal plane (0.55 µm) (xy) at the bone surface level. (**M**–**O**) These images show the enlarged insets indicated by a white box in I to K. Scale bars: 20 µm. (**P**) Left: Shows an enlargement of the areas indicated by a white box in M to O. Scale bar: 2 µm; arrows point to fibres positive for both F-actin and SEPT9. Right: Line scan through area marked by yellow dashed line in the lower left image. Colors: green, phalloidin/F-actin; red, IF staining of SEPT9; blue, dapi staining; grey, phase contrast; yellow, co-localization of phalloidin and SEPT9. Representative of 15 experiments.

Confocal imaging of bone resorbing OCs stained for SEPT9 revealed specific patterns correlating with distinct functional entities. Figure 3 shows a representative image (from a total of 15 experiments) of an OC engaged in bone resorption making a trench. Based on the F-actin staining (Fig. 3A) the orientation of the actin “ring” is crescent shaped defining the directionality of the resorptive front and hence the site of active bone demineralization and collagenolysis. Trailing behind this crescent ring is a long stretch of the cell body located inside the trench cavity. SEPT9 is found at many places in the cell in agreement with its many known roles from other cell types. A clear location of SEPT9 at the peripheral plasma cell-membrane is seen in patches reflecting very well what has been described^8,11,19,36,37^ from other cell types (Fig. 3B and C). However, the most conspicuous location of SEPT9 is its allocation to the ruffled border and adjoining plasmalemma that extends along the contour of the trench cavity (Fig. 3B–D). This pronounced concentration at sites where the OC is in close contact with eroded surfaces suggests a potential role for SEPT9 in bone resorption processes. For an animated view of Fig. 3C please refer to Supplementary Video S1. When looking at a confocal plane within the trench cavity, SEPT9 is preferentially located at the plasma membrane in contact with the bone at the sides of the trench cavity (Fig. 3E–H). Intense SEPT9 signals are observed especially where active resorption takes place at the leading edge of the cavity (Fig. 3I–O), which may suggest a distinct involvement at the ruffled border. But also on the bone surface, where early demineralisation and collagenolysis can be expected, a unique distribution of SEPT9 can be seen (Fig. 3M–O). On these surfaces F-actin fibres can be observed (Fig. 3M, O and P-upper left) and SEPT9 seems to be located along these actin fibres (Fig. 3N, O and P-centre left). Although a sign of co-localization can be seen as a yellow colour (Fig. 3P lower left, highlighted by arrows), a line scan (Fig. 3P right graph) suggests that they may not precisely overlap, but rather peak in the nadir of the other. This may be supported by reports suggesting that SEPT9 fibres bridge between different F-actin fibres^20,21^.

### SEPT9 and clathrin co-localization

**Figure 4:**
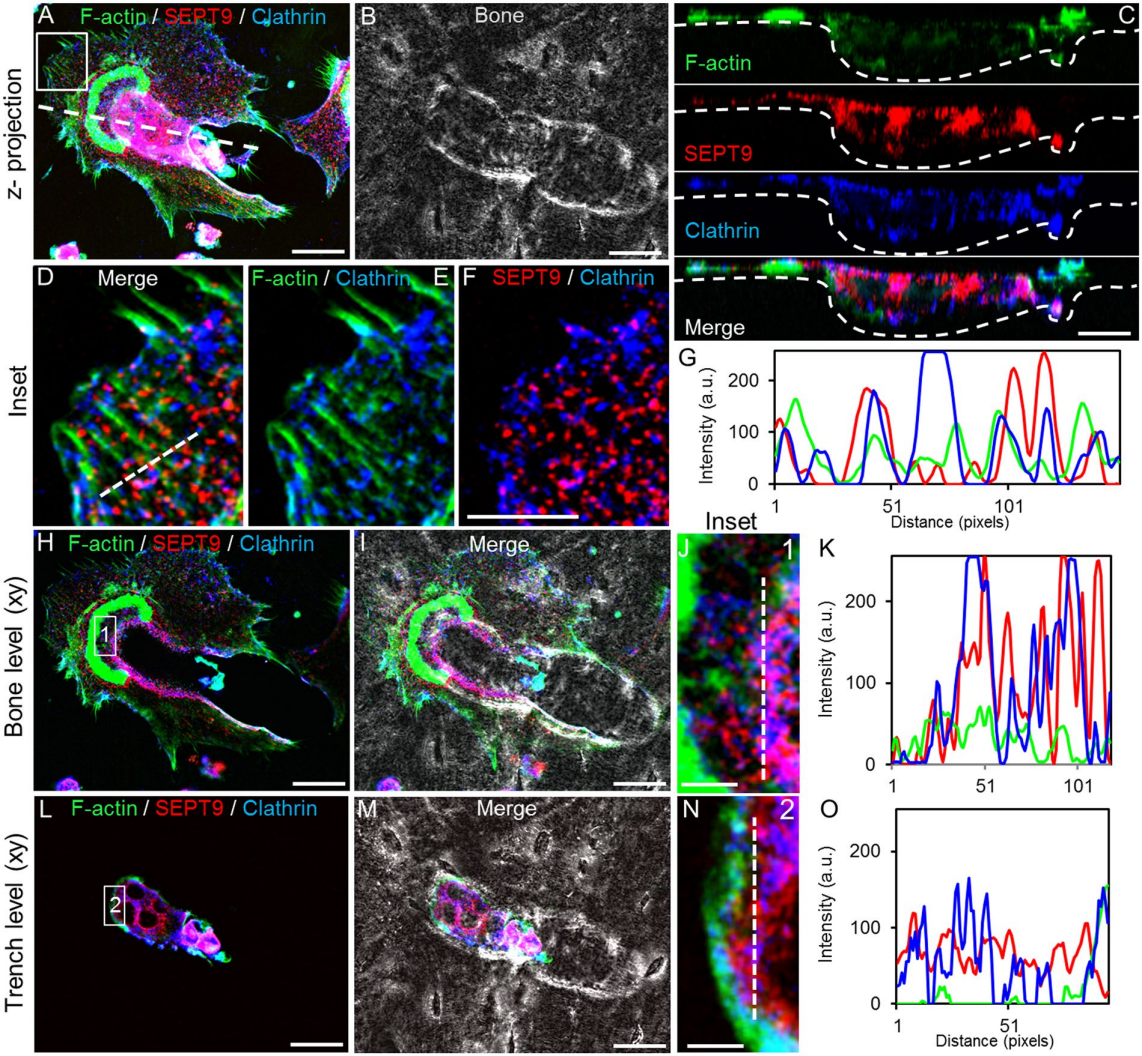
Trench-making human OC: Confocal IF detection of SEPT9 and clathrin show that they strongly co-localize at the sites of the OC engaged in active bone resorption and to those located within the boundaries of the trench. (**A**) 3D z-projection of trench resorbing OC. Scale bar: 20 µm. B) 3D z-projection using phase contrast to visualize the perimeters of the trench made by the OC shown in A. (**C**) Cross-section (xz) through the length axis of the OC and trench as indicated by a white dashed line in A. Scale bar: 10 µm. (**D**–**F**) A single confocal plane (0.55 µm) (xy) showing an enlargement of the pseudopod area indicated by a white box in A. within the cavity. Scale bar: 10 µm. (**G**) Line scan through the area marked by a white dashed line in D. (**H** and **I**) A single confocal plane (0.55 µm) (xy) at the bone surface level. Scale bar: 20 µm. (**J**) An enlargement of the area indicated by the white box 1 in H. Scale bar: 5 µm. (**K**) Line scan through the area marked by a white dashed line in J. (**L** and **M**) A single confocal plane (0.55 µm) (xy) at the trench level within the cavity. Scale bar: 20 µm. (**N**) An enlargement of the area indicated by the white box 2 in L. Scale bar: 5 µm. (**O**) Line scan through the area marked by a white dashed line in N. Colors: green, phalloidin/F-actin; red, IF staining of SEPT9; blue, IF staining of clathrin; grey, phase contrast; purple, co-localization of SEPT9 and clathrin. Representative of 5 experiments.

**Figure 5:**
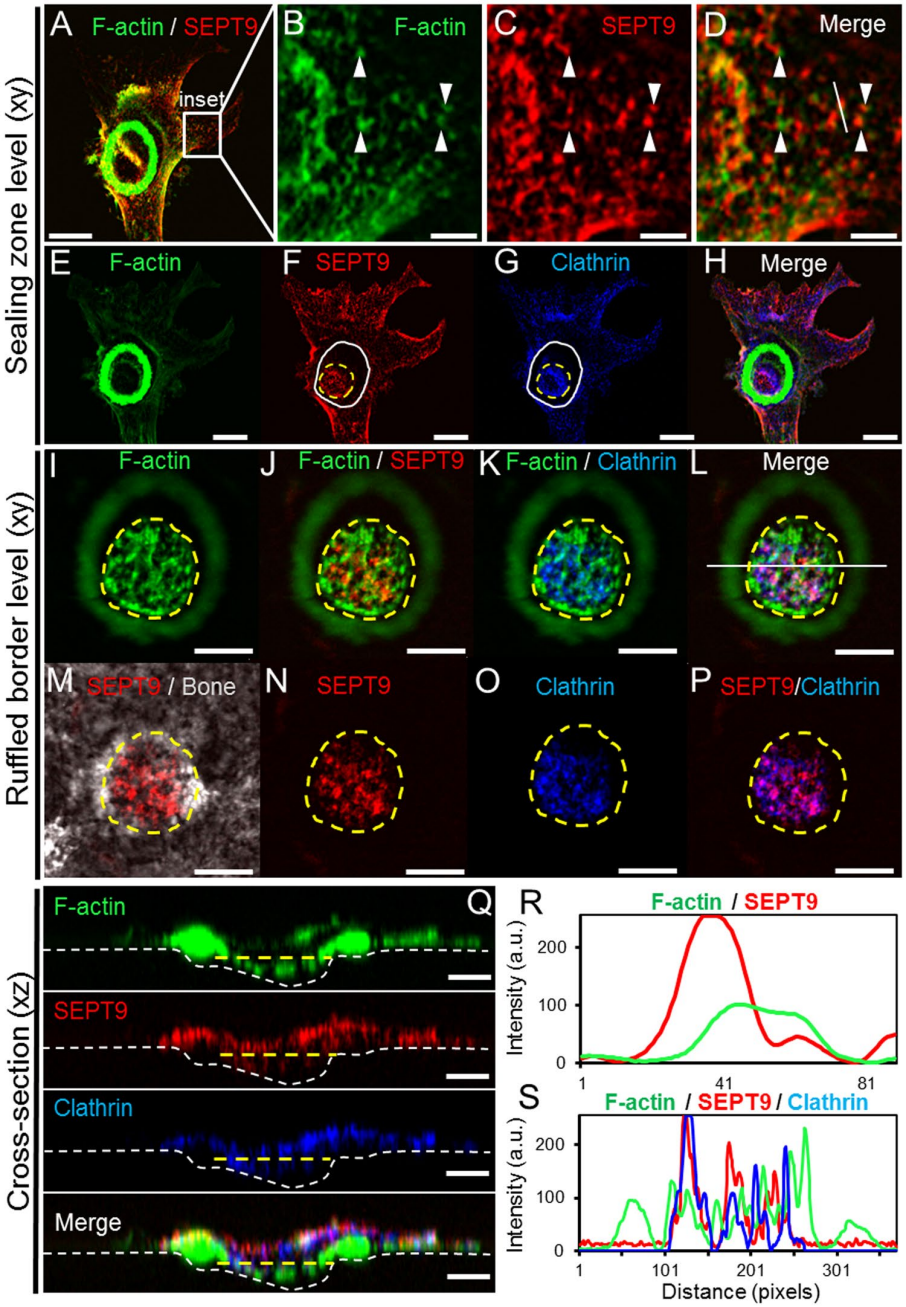
Pit-making human OC: Confocal IF detection of SEPT9 and clathrin. (**A**) A single confocal plane (xy) at the level of the sealing zone. Scale bar: 20 µm. (**B**–**D**) A single confocal plane (0.55 µm) (xy) showing an enlargement of the area indicated by a white box in A. Scale bars: 5 µm. White arrow heads point to F-actin and SEPT9 fibers that seem to be attached to one another. (**E**–**H**) A single confocal plane (xy) a few planes deeper than the one shown in A, at the transition between the bone surface and the beginning of the cavity. Scale bars: 20 µm. The white solid line marks the inside of the sealing zone while the yellow dashed line indicates the perimeter of the cavity. (**I**–**P**) Show different representations of the same confocal plane (xy) within the pit-cavity. Scale bars: 10 µm. (**Q**) Cross-section (xz) through the OC and pit as indicated by a white line in L. Scale bar: 10 µm. (**R**) Line scan through the area marked by a white line in D. (**S**) Line scan through the resorptive zone as indicated by a yellow dashed line in Q. Colors: green, phalloidin/F-actin; red, IF staining of SEPT9; blue, IF staining of clathrin; grey, phase contrast; purple, co-localization of SEPT9 and clathrin. Representative of 5 experiments.

During bone resorption, clathrin-dependent endocytosis is involved in uptake of degraded bone components^38–41^. At low magnification co-localization between SEPT9 and clathrin can be seen within the limits of the trench cavity (Fig. 4A). However, when zooming into the resorption cavity both in the xz- (Fig. 4C) and xy-plane (Fig. 4H–J and L–N) the extent of co-localization is largely overlapping or interchanging (Fig. 4K–O). The most prominent locations of both factors are clearly at the edges of the resorption cavity and towards the bottom of the cavity possibly at the base of the ruffles. A characteristic organization of clathrin and SEPT9 along F-actin stress fibres is also seen in lamellipodia (Fig. 4D–G). Such SEPT9 filaments connected with F-actin fibres are also observed in cell protrusions of an OC making a pit (Fig. 5A–D and R). Also at the level of the ruffled border such interconnections between SEPT9 and F-actin can be seen (Fig. 5J). A line scan through both areas (Fig. 5R and S) highlights that the two types of structures are adjacent. As it was seen for the trench-making OC in Fig. 4, SEPT9-containing fibrils strongly co-localize with clathrin within the ruffled border region of the pit-making OC as noted by the purple colour (Fig. 5J, K, L, N, O and P). On the line scan plot an overlap of several SEPT9 and clathrin-containing structures is also clear (Fig. 5S). Also in the profile view of the OC within the pit it can be seen that clathrin and SEPT9 have the same distribution within the cell (Fig. 5Q).

### SEPT9 forms complex fibrillar structures with microtubules

**Figure 6:**
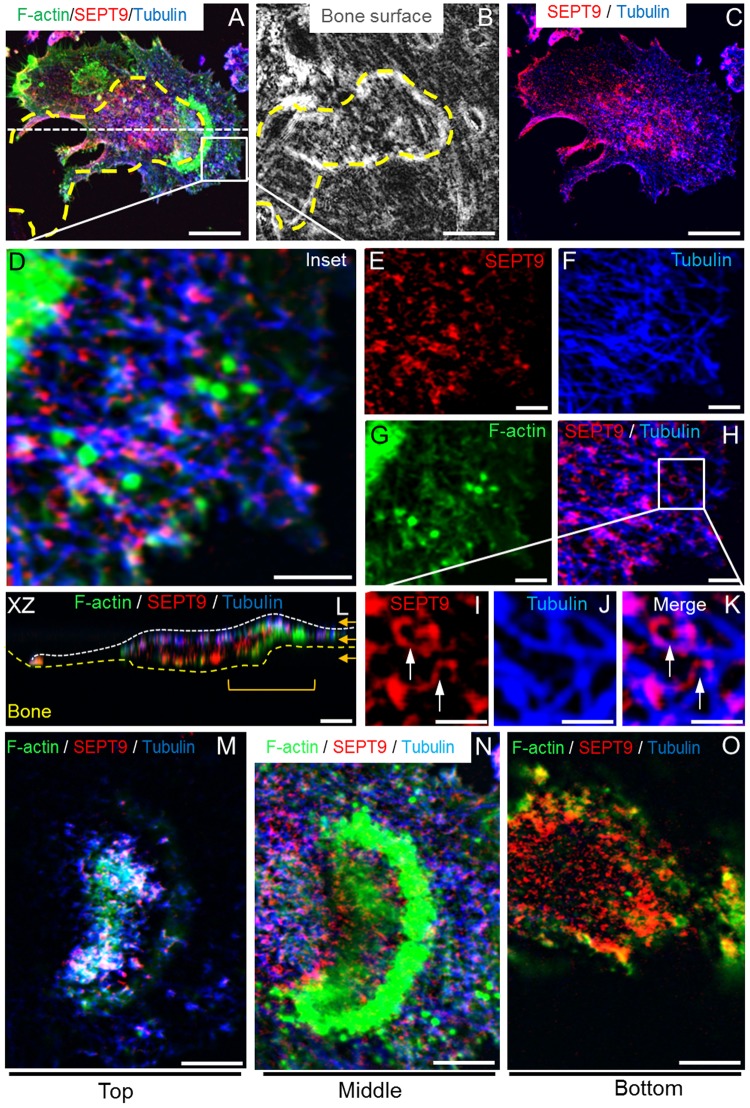
SEPT9-containing fibrils strongly co-localize with the microtubular network in human bone resorbing OCs. (**A** and **C**) 3D z-projection of trench-making OC. Scale bars: 20 µm. (**B**) Phase contrast image of trench cavity made by the OC shown in A and C. Yellow dashed line indicates the perimeter of the trench. Scale bar: 20 µm. (**D**–**H**) Zoom in on the highlighted zone in A. Shown is one confocal z-plane (0.55 µm) with the indicated staining. Scale bars: 5 µm. (**I**–**K**) Zoom in on the highlighted zone in H. Shown is one confocal z-plane with the indicated staining. White arrows point to examples of joined microtubule and SEPT9 containing filaments. Scale bars: 2.5 µm. (**L**) Profile view through the OC along the axis indicated by a white dotted line in A. (**M**–**O**) Show selected z-planes through OC as indicated by the orange arrows in L, representing the top middle and bottom fractions of the OC, respectively. Scale bars: 10 µm. Colors: green, phalloidin/F-actin; red, IF staining of SEPT9; blue, IF staining of α-tubulin; grey, phase contrast; purple, co-localization of SEPT9 and α-tubulin. Representative of 5 experiments.

From other cell types it is known that especially SEPT9 mediates the binding of heterogenic septin filaments to microtubules regulating e.g. direction of vesicular transport^7,11,19^. Confocal IF images of SEPT9, F-actin and microtubules clearly show that such interactions can also be observed in OCs in different compartments of the cell (Fig. 6A and C). From Fig. 6A and C it is evident that there are unique structures formed between SEPT9 and microtubules both at pseudopods, uropods and in the cell compartments involved in bone resorption. The structures formed in pseudopod-like cell compartments are shown in Fig. 6D–K. When enlarged, it is clear that horizontal SEPT9 fibrils seem to run along microtubules and are apparently at least 2.5 µm long (Fig. 6I–K). When focusing on the areas of the cell at the resorptive site there is still a high degree of association with microtubules at the upper part of the cell (Fig. 6M, upper orange arrow in Fig. 6L) and at the level of the leading actin “crescent” (Fig. 6N, centre orange arrow in Fig. 6L). At those levels extended horizontally oriented SEPT9 containing fibrils are still seen. However, once getting below the bone surface and into the resorption cavity (Fig. 6O, lower orange arrow in Fig. 6L) microtubules are mostly absent and SEPT9 containing fibrils are no longer as prominent. At this level they become more dotted suggesting that their orientation may be rather perpendicular than parallel to the bone surface.

### Conditional knock-out of Sept9 in male mice results in a mild osteopetrotic phenotype

**Figure 7:**
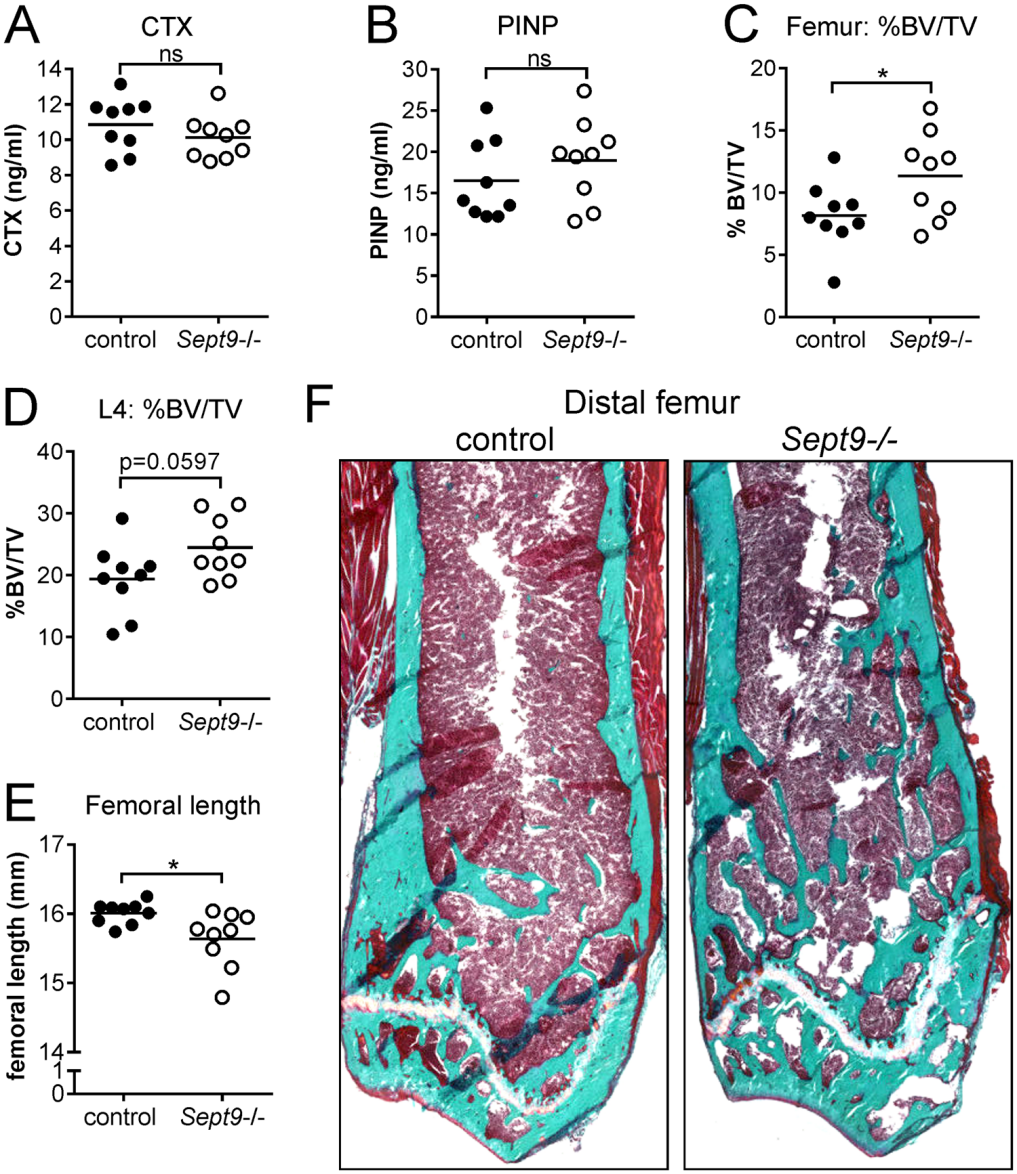
Conditional knock-out of the *Sept9* gene in adult male mice results in a mild osteopetrotic phenotype. Knock-out was induced at 10 weeks of age and mice were sacrificed at week 30. Fasting blood samples were analyzed for levels of bone-turnover makers: (**A**) CTX (p = not significant) and (**B**) PINP (p = not significant). The trabecular bone volume per tissue volume was determined on the (**C**) left femora (*p = 0.0445) and (**D**) L4 vertebrae (p = not significant) while (**E**) the length was determined on the right femora (*p = 0.0272). (**F**) Representative images of histological Masson-Goldner trichrome stained tissue sections of the distal femur of a control and *Sept9*^−/−^ mouse. The total length of the images represents 5.3 mm. (A–D) Statistics: unpaired t-test and (E) Statistics: unpaired t-test with Welch’s correction; two-tailed. n = 9 control and n = 9 *Sept9*^−/−^.

In order to address whether SEPT9 plays a significant role in OCs *in vivo* we deleted the *Sept9* gene in OCs of mice homozygous for the conditional *Sept9*^*cond*^ allele^36^. As a full knock-out of *Sept9* is embryonically lethal, we crossed these mice with *Mx-cre* transgenic mice, which upon induction with interferon express CRE recombinase predominantly in the hematopoetic lineage and the liver^36,42^. Thus, with respect to bone homeostasis, *Sept9* will be deleted in OCs but not in osteoblasts. We induced the Cre-recombinase when the animals were 10 weeks old and sacrificed them when they were 30 weeks old. At this time-point the CTX-levels in the plasma were slightly but not significantly reduced in the *Sept9*^−/−^ mice (Fig. 7A), while PINP-levels were slightly but not significantly elevated (Fig. 7B) compared to control mice. These markers only represent a brief snapshot in time, but have a cumulative effect over time. Therefore, it is particularly interesting that the persistent loss of *Sept9* over a period of 20 weeks resulted in significant elevation of the distal femoral trabecular bone volume per tissue volume (BV/TV) from 8.2% to 11.4% (Fig. 7C) while cortical thickness at the mid-diaphysis was unaffected (mean of 197 and 204 µm, respectively, p = 0.60, data not shown). Representative images of distal femora from control and *Sept9*^−/−^ mice are shown in Fig. 7F. Furthermore, the L4 BV/TV increased from 19.4% to 24.5% but only reaching near-significance (Fig. 7D). We also found that the lengths of femora in male control mice (Fig. 7E) were very homogenous and had a mean length of 16.0 ± 0.16 mm. In contrast, *Sept9*^−/−^ mice were much more heterogenic and had a mean length of 15.4 ± 0.41 mm. Although the mice were 10 weeks old when the knock-out was induced, we still observed a significant drop in femoral length in the *Sept9*^−/−^ animals compared to the controls, a strong indicator of reduced OC activity. Please note that Fig. 7F clearly shows a narrow but active growth plate containing hypertrophic chondrocytes at 30 weeks of age supporting continued growth even beyond 10 weeks of age. The activity levels of tartrate-resistant acid phosphatase (TRAcP) in the plasma (a marker reflecting the number and nuclearity of OCs *in vivo*) were not different between the control and *Sept9*^−/−^ groups (mean absorbance of 0.321 and 0.320, respectively, p = 0.98, data not shown) and the OC surface *in situ* was unaffected (Supplementary Fig. S2), suggesting that osteoclastogenesis was not affected by *Sept9* deletion. Thus, our data suggest that there is a function of SEPT9 in OC resorption *in vivo*. In addition, multiple linear regression analyses and likelihood ratio tests (based on the *in vivo* data) show that *Sept9* deletion, was the strongest significant predictor of the dependent variables (BV/TV (femoral and L4) and femoral length) (data not shown).

## Discussion

Septins were identified in the 1970s and have since then been found to be expressed and play roles in various organisms and cell types. In humans, mutations in septins are known to play a role in cancer and mutations in the *SEPT9* gene can cause hereditary neuralgic amyotrophy^16–18^. Here we show that: 1) during differentiation of human OCs the *SEPT9* gene is expressed resulting in a variety of different SEPT9 splice variants; 2) septins are involved in the bone resorptive activity of human OCs; 3) a conditional knockout of murine *Sept9* in the hematopoietic lineage results in increased levels of femoral and lumbar trabecular bone volume and a femoral growth retention; 4) immunolocalizations of SEPT9 in actively resorbing OCs document distinct localization at several sites and preferentially at the ruffled border and membranes juxtaposed to eroded bone surfaces; 5) SEPT9 is associated to the cytoskeletal elements of the OC such as F-actin and α-tubulin; 6) SEPT9 encircles clathrin-rich patches. Thus, we conclude that SEPT9 is expressed in human and murine OCs and that septins are directly involved in facilitating osteoclastic bone resorption and that SEPT9 may play a special role in this process.

The *SEPT9* gene is expressed as numerous splice variants both in mice^43^ and humans^26^. The SEPT9 protein resulting from these splice variants varies in predicted MW from around 38 to 69 kDa^26^, but some have almost identical MWs. In a series of papers it was reported that expression of these variants of SEPT9 vary between different tissues^26,27,29^ as well as in cancer^28,30^. Our results show that the expression-pattern of the variants changes during differentiation stages from monocyte to macrophage to OC. After a seven day exposure to RANKL two SEPT9 variants with an apparent MW of 58 and 37 kDa dominate while three other variants (68, 42 and 40 kDa) are less frequent. We are not able to say precisely which splice variants they are, but from the existing literature it is likely that the variants of 58 and 68 kDa contain the N-terminal domain but not the smaller products^7,26,37^. The N-terminal domain was found to mediate the binding to structures such as microtubules and actin^7,19–21^. When isoforms lacking this domain or carrying mutations are overexpressed the organization of these cytoskeletal structures is altered^7,19,44^. This suggests that the isoforms may have different functions and that their dynamics and turn-over could be part of the functional regulation. However, more studies are required to precisely identify the different splice variants expressed in OCs.

The importance of septin turnover for their function in OCs is evident from our experiments using FCF and the *Sept9* knock-out mice. FCF has been shown in several studies to stabilize septin fibres by specifically preventing their turnover^31,32^ possibly by interfering with the GTP binding site^45^. We found that this drug had a pronounced and reproducible inhibitory effect on osteoclastic bone resorption with an IC50 of around 20 µM. FCF has previously been reported to inhibit mitosis, exocytosis, microtubule protrusions and cell migration^12,32,35,46,47^. Especially cell migration is interesting because it is known to be important for proper bone resorption. When OCs are in pit-mode they need to move between periods of active resorption and in trench-mode the OCs move while they resorb^25^. Thus, FCF may allow an OC to make an initial pit, but prevent it from making an additional one. However, OCs may be prevented from making a trench because this by definition demands simultaneous movement and resorption. The consequence could be that OCs stop resorbing after making an initial pit, which is a prerequisite for most OCs (80%) making a trench^25^. This might explain why the IC50 of trench surface/BS is significantly and reproducibly lower than for pit surfaces.

The conditional knock-out of *Sept9* in the hematopoietic lineage of mice resulted in a mild osteopetrotic phenotype both regarding BV/TV in the femur and L4 as well as a mild growth defect resulting in shorter femora. This suggests that under *in vivo* conditions SEPT9 plays a role in the bone resorptive activity of OCs since both of these outcomes demand that OCs are negatively affected by the knock-out. A possible explanation for the observed bone phenotype could be an effect on OC number, osteoclastic bone resorption or both. Since end-point analyses of plasma TRAcP-levels *in vivo* and OC surface *in situ* were not different, it suggests that the knock-out foremost affects the resorptive activity of OCs. However, in order to address this conclusively it would demand to perform *in vitro* analyses using bone marrow OC cultures from these mice. Such experiments are planned for an upcoming study. In addition, it may be mentioned that it is surprising to observe an effect on femoral length when the animals were 10 weeks old at induction since most of the growth occurs up to the 10^th^ week of age in mice. However, residual growth still occurs beyond this age in these male mice, which is supported by the fact that a narrow but active growth plate can still be detected even when the animals are 30 weeks old (Fig. 7F).

The effect of the *Sept9* knock-out was mild, but there may be several reasons for this: 1) the animals were 10 weeks old when the knock-out was induced and they were not challenged by any other treatment (such as orchiectomy). Dramatic differences could therefore not be expected. 2) SEPT9 is only one element in the hetero-octamer that septins form. It belongs to the SEPT3 group that also includes Septin 3 and Septin 12. When a gene from this family is deleted it may therefore partly be replaced by another member of the SEPT3 family^48^, which may explain the mild phenotype. In contrast to this, mutations in the SEPT9 gene affecting the function of SEPT9 can be expected to have a more severe effect, such as in hereditary neuralgic amyotrophy, because the defective protein can still occupy its space in the hetero octamer^18^. We are aware that this mouse model has its limitations especially because the Mx1-Cre promotor induces knock-out throughout the hematopoietic lineage and thereby not only in OCs. Effects from other cells than OCs, which contribute to the phenotype, can therefore not be categorically ruled out. However, currently there are no other inducible *Sept9* knock-out mouse models available that can delete *Sept9* in OCs. Nevertheless, the present study should now encourage scientists to generate an e.g. cathepsin K inducible Cre-mouse and thereby gather more critical *in vivo* evidence.

Further support for a role of SEPT9-containing septin filaments in the bone resorptive processes are indicated by the confocal analyses of SEPT9 localization. Especially the images in Figs 3 and 4 highlight that SEPT9 is particularly abundant at sites where the edges of trench cavities (both front and sides) force the actin cytoskeleton of the OC to bend. Exactly at these sites the cytoskeleton is put under special strain in trench-making OCs because the sharp angle between bone surface and cavity wall coupled with the movement of the trench-making OC must generate mechanical strain both on the cytoskeleton and the membrane. This may require the stabilizing function of septins to a larger extent than in pit-making OCs. An overlap of actin and SEPT9 at these sites is interesting because septins have been reported to promote F-actin stability, bundling, bending and to cross-link different cytoskeletal structures giving them the necessary stability^7,19–22,34,49^. Similar stability may be given to the membranes themselves by septin filaments alike the septin “corset” containing SEPT9 in crawling T-cells^50^.

Although septins in general have been reported to stabilize cytoskeletal structures they have also been reported to facilitate their turnover enabling movement^20,30,35,36,44,50^. Although this may sound paradoxical it is thought to be because of the different roles of the splice variants. Long variants of SEPT9 contain an extended N-terminal domain enabling binding to e.g. actin and microtubules, while shorter variants in general lack this domain^7,19,21,26,37^. This can either stabilize or destabilize the cytoskeleton, respectively, eventually enabling migration^30,44^ and at least in some situations the knock-out of *SEPT9* can have the same effect^20,36^. Thus, a dynamic exchange of long and short splice variants could be essential to ensure continued activity of OCs. This may be particularly relevant at the ruffled border. The ruffles are supported by an actin and microtubule network that physically supports the protrusion, but also facilitates transport of vesicles for exo- and endocytosis. Septins (including SEPT9), were found to facilitate membrane protrusions^10,44,47^ by guiding microtubule growth at the plus-end^35^ towards the bottom of the resorption cavity^51^. Thus, septins may be involved in promoting the dynamics of ruffles and their change in shape and location.

Septins are also implicated in vesicle transport^9,11,12^ and membrane trafficking^52^. Clathrin is a prominent factor of endocytosis for osteoclastic resorptive activity with a unique location at the ruffled border^38–40,53^, more specifically at the base of the ruffles^53^. Our confocal analyses of IF stained clathrin showed that in pit-making OCs the clathrin signal at the ventral membrane of the OC was most prominent at the central ruffled border respective to the actin ring (Fig. 5O and Q), whereas in trench-making OCs the signal starts at the leading edge of the ruffled border and stretches far beyond the lagging actin ring within the resorbed cavity (Fig. 4C). In both types of OCs, clathrin was found to coat more or less the entire basolateral and dorsal membrane. This is in good agreement with an earlier report^40^. In order to interpret the organisation of SEPT9 signals, knowledge from other cell types should be considered. Septins are for example described to form ring-like structures at the base of cell extensions and act as a diffusion barrier^1^. At the same time clathrin coated invaginated vesicles form in a spatially very restricted manner^38,53^. In order for this to occur effectively it can be speculated that septins in general and SEPT9 in particular may facilitate this spatial restriction by generating a diffusion barrier. A double IF staining for both clathrin and SEPT9 has to our knowledge not been done before. But by inspection of images such as shown in Fig. 4J,K,N and O as well as Fig. 5P and S it is possible that SEPT9-containg filaments might form a structure around clathrin rich zones within the ruffled border. Their possible location at the base of ruffles could also explain why the signal from both factors is slightly lifted from the bottom of the cavities both in pit- and trench-making OCs (Figs 4C and 5Q). The formation of diffusion barriers may facilitate a faster and more efficient execution of endocytosis and given that trench-making OCs remove bone roughly 3-times faster than those making pits^25^, it could explain why trench-making OCs are more sensitive to FCF than pit-making OCs (Fig. 2H).

In conclusion, we have shown that septins in general and possibly SEPT9 in particular play a role in facilitating efficient bone resorption by OCs. Septins and SEPT9 have key roles in cytoskeletal stability and turnover, directing vesicular transport, endo- and exocytosis, stabilizing membrane structures and as diffusion barriers. Since these are all processes that are central for osteoclastic bone resorption we find that it is important to further investigate the role and importance of septins in both murine and human OC model systems. This may facilitate a better understanding of how the complicated processes during osteoclastic bone resorption are coordinated and positioned, and how they may be better targeted by drugs to regulate the resorptive activity.

## Methods

### *In vitro* generation of human OCs

CD14^+^ monocytes were purified from anonymous human blood donors (informed consent, approved by the local scientific ethical committee, 2007-0019) by centrifugation through Ficoll-Paque (Amersham, GE Healthcare, Little Chalfont, UK) and subsequently isolated by immunomagnetic separation following the procedure described in^54^. In brief, peripheral blood mononuclear cells were suspended in 0.5% BSA in PBS (with 2 mM EDTA) and CD14^+^ cells were purified using BD IMag Anti-Human CD14 Magnetic Particles – DM (BD Biosciences, San Jose, CA, USA) according to the instructions by the supplier. CD14^+^ monocytes were seeded at a density of 6.7 × 10^4^ cells/cm^2^ and differentiated into mature OCs over 9 days with MCSF and RANKL (both from R&D Systems, Minneapolis, MN, USA) as previously described^25,55,56^.

### Western-blot analyses

Western-blots were performed with cell lysates obtained from five different stages of OC differentiation (please refer to figure legend). Cells were lysed and lysates were stored at -20 °C in aliquots. Cell lysates and prestained molecular weight markers were separated on Criterion precast 10% Bis-Tris gels. The same amount of protein extract was loaded in each lane and gels were run according to descriptions by the supplier (BioRad, Hercules, CA, USA). Proteins were blotted with transfer buffer (10 mM NaHCO_3_ and 3 mM Na_2_CO_3_) onto a nitrocellulose membrane (BioRad), blocked with TBS + 0.1% TWEEN20 (TBST) + 3% (w/v) BSA for 1 h, washed, and incubated with the primary antibody (polyclonal rabbit-αhSEPT9 antibody (10769-1-AP, Proteintech, Manchester, UK)) or polyclonal rabbit-αhCatK antibody (ab49893, Abcam, Cambridge, UK)) in TBST + 3% BSA for 16 h at 4 °C. The gel was washed, incubated with HRP-coupled secondary antibody (anti-rabbit antibody (ECL WB system, RPN 2108, GE Heath Care)) in TBST + 3% BSA for 1 h, washed and then developed using ChemiDoc Imaging System (Bio-Rad). Subsequently, gels were stripped (0.1 M glycine, 20 mM magnesium acetate and 50 mM potassium chloride, pH 2.2) for 2 × 10 min at room temperature, washed and incubated with monoclonal mouse-αhβActin antibody (clone AC-74, Sigma-Aldrich, St. Louis, MO, USA) and processed as aforementioned.

### Q-PCR

Cells were lysed and RNA was isolated using the Trizol Plus RNA Purification kit (Invitrogen, Carlsbad, CA, USA) as described in^57^. cDNA was generated using 500 ng RNA and the iScript kit (BioRad) followed by Q-PCR, performed using the TaqMan approach and run on a Realtime PCR machine (79000HT, Applied Biosystems, Foster City, CA, USA). Triplicates of the PCR-reaction were performed for each of the triplicate cultures per condition. Each Q-RT-PCR run was normalized to a cDNA standard curve. ALB and GUS were used as reference genes, and the expression levels of the genes of interest were adjusted relative to the average expression levels of ALB and GUS. All TaqMan primer/probe sets were used according to instructions by supplier (Applied Biosystems) (Primer/Probes: GUS, Hs99999908_m1; ABL, Hs00245443_m1; *SEPT9*, Hs00246396_m1; CATK, Hs00166156_m1).

### Bone resorption assays

Mature OCs (7 days with RANKL) were detached using accutase (Biowest, Nuaillé, France) and reseeded on 0.4 mm thick bovine bone slices (Boneslices.com, Jelling, Denmark) at a density of 100,000 cells/bone slice. OCs were cultured in the presence of 10% FCS, 25 ng/mL MCSF, RANKL and when indicated, the septin inhibitor, forchlorfenuron (FCF, Sigma-Aldrich). After 3 days of incubation, the media was stored at −20 °C for later TRAcP analyses^54^. CellTiter-Blue Cell viability Assay (Promega, Fitchburg, Wisconsin, USA) was used according to instructions by the supplier.

Resorption events were visualized with toluidine blue staining as previously described^58^ and analysed by light microscopy for the percentage of eroded surface and the number of resorption events. The percentage of eroded surface was determined through light microscopy by analysing the entire bone surface with a 10x objective through a 100 point grid as described in^55,59^. The number of resorption events was determined by analysing the entire bone surface through light microscopy equipped with a 20x objective. In these analyses, the two types of resorption patterns (pits and trenches) were distinguished as described in^59^. In order to evaluate the number of multinucleated (≥2 nuclei) TRAcP-positive (TRAcP^+^) cells present on the bone slice, cells were stained for TRAcP using the Leukocyte Acid Phosphatase kit (Sigma-Aldrich), and the number of cells was determined by analysing the entire bone surface using light microscopy. All quantifications were performed blinded.

### Immunofluorescence

Mature OCs (7 days with RANKL) were after an additional three days of resorption on bone slices (0.2 mm; Boneslices.com) washed in PBS, fixed in 3% paraformaldehyde and 2% sucrose for 15 min at room temperature, washed, blocked, permeabilised (PBS, 0.5% BSA, 0.05% saponin), incubated with the primary antibodies in different combinations (polyclonal rabbit anti-human Septin 9 (10769-1-AP, Proteintech), monoclonal mouse anti-human Clathrin (ab2731, Abcam), mouse monoclonal anti-human α-tubulin (clone DM1A, T9026, Sigma-Aldrich)) for 60 min, washed, and incubated with the secondary antibody goat anti-rabbit AF647 (111-605-144, Jackson Labs, West Grove, PA, USA) or goat anti-rabbit sAb AF568 (A12379, Invitrogen) or goat anti-mouse sAb AF568 (A11019, Invitrogen) or goat anti-mouse AF647 (115-605-205, Jackson Labs). Finally, cells were stained for F-actin using phalloidin AF647 (A22287, Invitrogen) or phalloidin AF488 (A12379, Invitrogen). Bone slices were mounted with ProLong Gold containing DAPI (Invitrogen). Confocal images were obtained using an Olympus Fluoview FV10i microscope (Olympus Corporation, Shinjuku, Tokyo, Japan)(resolution xy-plane: 0.1 to 0.2 µm/pixel; z-plane: 0.55 to 1 µm/slice) and images were processed using Imaris version 7.6.5 (Bitplane AG, Zurich, Switzerland). Images were deconvolved using the Iterative Deconvolution 3D plugin of ImageJ (National Institutes of Health) with five iterations. Intensity profile graphs of images were calculated by a custom pixel-based Linescan Intensity Profiler (MATLAB, The Mathworks, Inc., Natick, MA, USA) as previously described^51^. Images shown in Figs 3–6 are representative examples from at least 15 experiments (different blood donors). SEPT9 was immunolabeled in all 15 experiments while co-staining for clathrin or microtubules each were done in 5 experiments. Each experiment included at least 10 good quality images.

### Mouse breeding

C57Bl/6 J mice homozygous for the conditional *Sept9* KO *Sept9*^*TM2-EMFU*^ allele^36^, in this article termed *Sept9*^*cond*^, were mated with transgenic C57Bl/6 J mice hemizygous for the *Tg(Mx1-cre)1Cgn* transgene, which codes for CRE recombinase under the control of an interferon dependent promoter. Upon induction, the transgene is predominantly expressed in the liver and the hematopoetic system^42,60^. In this article this transgene is termed *Mx-cre*. Backcross of hemizygous *Mx-cre* mice that were heterozygous for *Sept9*^*cond*^ resulted in *Mx-cre* transgenic, homozygous *Sept9*^*cond/cond*^ mice. Mating of these with non-transgenic homozygous *Sept9*^*cond/cond*^ mice was used to produce experimental animals. The 50% not bearing the *Mx-cre* transgene, served as control animals. Expression of CRE recombinase results in deletion of exon 2-5 of the *Sept9* gene. The official symbol for the deleted allele is *Sept9*^*TM2*.*1-EMFU*36^, but in this article it will be termed *Sept9*^−/−^.

### Induction of CRE expression and housing

At the age of 10 weeks, male mice were injected eight times with one day interval with 12 µg double strand pIpC RNA/g body weight to induce endogenous interferon production. Mice were weighed before each injection. Independent of the presence of the *Mx-cre* transgene, mice lost between 10 and 20% weight after the first injection but recovered to the original weight during the 14 day injection period and at sacrifice there was no weight difference between the two groups (controls 33.7 g ± 4.9 and *Sept9*^−/−^ 35.4 g ± 4.7, p = 0.5026). The animals were housed at 20 °C with a 12/12 h light/dark cycle. The animals had free access to standard mice chow (1324, Altromin, Brogaarden, Denmark) and tap water.

### Tissue preparation and bone biomarker analyses

Animals fasted overnight and were, 138 days after the initial plpC RNA injection, euthanized by anesthesia (IsoFlo Vet, Orion Pharma Animal Health) and the heart was removed. Blood was obtained from vena cava inferior, plasma isolated and stored at −80 °C and was later analysed for CTX and PINP according to instructions by the supplier (RatLaps CTX and Rat/Mouse PINP, IDS, Boldon, Tyne & Wear, UK). The femora, tibiae, and lumbar vertebrae were isolated and carefully cleaned from all soft tissue. The left femora and L4s were immersion-fixed in 70% ethanol. Furthermore, the liver was removed, weighed, and frozen at −80 °C. Successful induction of Cre activity by the pIpC treatment was verified by the presence of the *Sept9* KO allele in liver DNA, as a strong correlation between Cre activity in liver and bone marrow has previously been reported in these mice^42,60^.

### Embedment, sectioning and histomorphometry of mouse bones

The mouse femora and L4 were ethanol fixed and embedded undecalcified in methyl methacrylate (MMA), while L1-2 were formalin fixed, decalcified and embedded in paraffin^61^. Sequential 7-µm-thick vertical MMA sections were cut of femora and L4s and stained with Masson-Goldner trichrome. Three sections, separated by 8-10 sections, intercepting the central part of the marrow cavity were selected, scanned, and used to estimate the trabecular bone volume (BV/TV) using a point-grid analysis. The estimate was performed within the complete marrow cavity of the L4 and in marrow cavity 0-5 mm from the distal growth plate of the femora. Sequential 3.5-µm-thick vertical paraffin sections were cut of L1-2 vertebrae and histochemically stained for TRAcP activity^61^. A single section from the centre of each L1-2 vertebrae was scanned and used to estimate osteoclast surface per bone surface (Oc.S/BS) using a Mertz grid^62^. Both endo-cortical and trabecular surfaces were counted and pooled obtaining at least 292 hits per section (min/max: 292/1549). The investigator was blinded during all analyses. Femoral length was determined by using a caliper.

### Statistical analyses

All statistics shown on graphs were performed using GraphPad Prism software, version 5 (GraphPad software, San Diego, CA, USA), and statistical significance was defined as p < 0.05. The D’Agostino & Pearson omnibus test was used to test for normality. Multiple linear regression analyses were performed using STATA version 12.1 (STATACorp, Collage Station, TX, USA). Please refer to figure legends for further detail.

### Study approval

All methods were carried out in accordance with relevant guidelines and regulations. The use of human blood donors was approved by The Scientific Ethical Committee for the Region of Southern Denmark with approval number 2007-0019. All subjects were 18 years or older and informed written consent was obtained from all subjects. The animal study was done in accordance with the EU Directive 2010/63/EU for animal experiments and was approved by the Danish Animal Experiments Inspectorate (2015-15-0201-00517).

## Electronic supplementary material

Supplementary Video S1

Supplementary information

## Data Availability

The datasets generated during and/or analysed during the current study are available from the corresponding author on reasonable request.
