# Supplementary material for: Septins are critical regulators of osteoclastic bone resorption
Source: Sci Rep. 2018 Aug 29;8:13016. doi: 10.1038/s41598-018-31159-1 (PMC6115361; doi:10.1038/s41598-018-31159-1)
Supplement: Supplementary file 2 — Supplementary information [file 41598_2018_31159_MOESM2_ESM.docx]

Septins are critical regulators of osteoclastic bone resorption

Anaïs MJ Møller, Ernst-Martin Füchtbauer, Annemarie Brüel, Thomas L Andersen, Xenia G Borggaard, Nathan J Pavlos, Jesper S Thomsen, Finn S Pedersen, Jean-Marie Delaissé and Kent Søe

Supplementary Information

**Supplementary Figure S1**

**Full unedited gel for Figure 1C**

SEPT9 β-actin


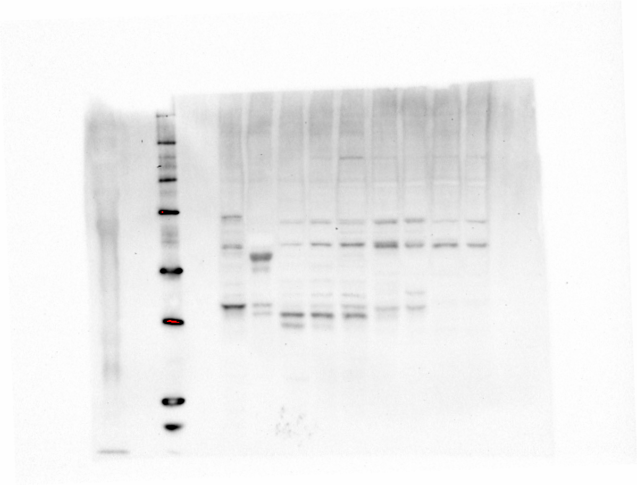

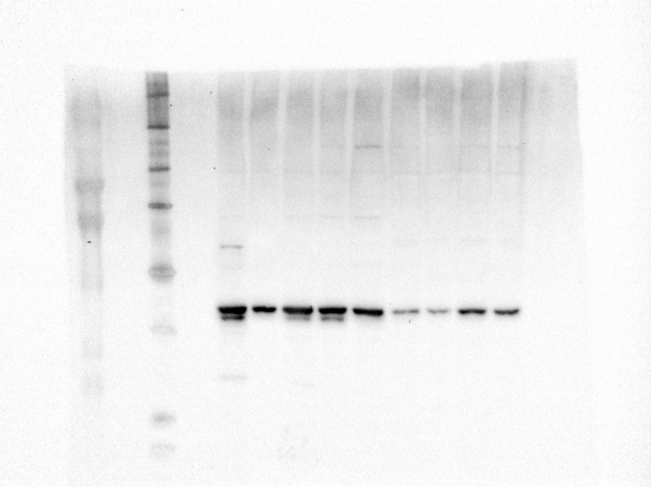


**Full unedited gel for Figure 1D**

CatK β-actin


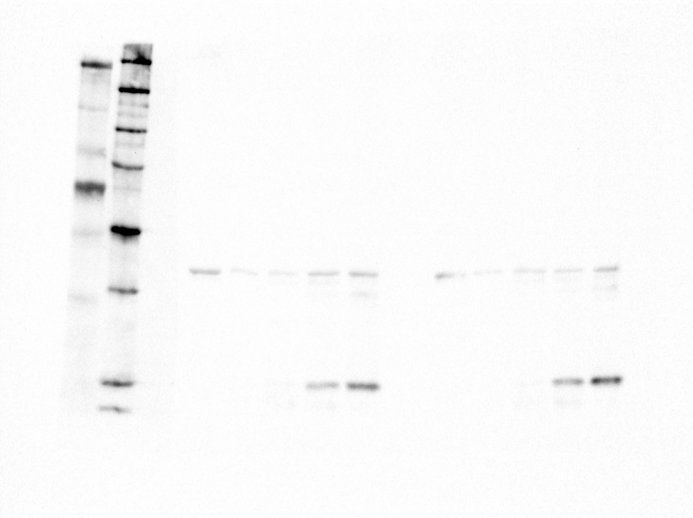

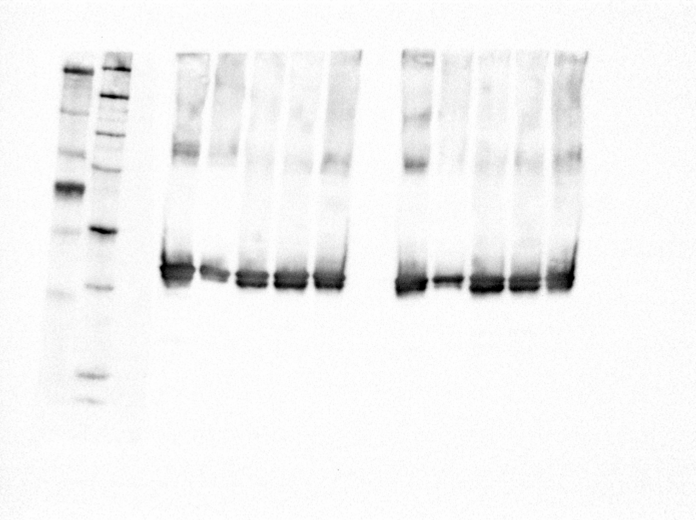


**Supplementary Figure S2**


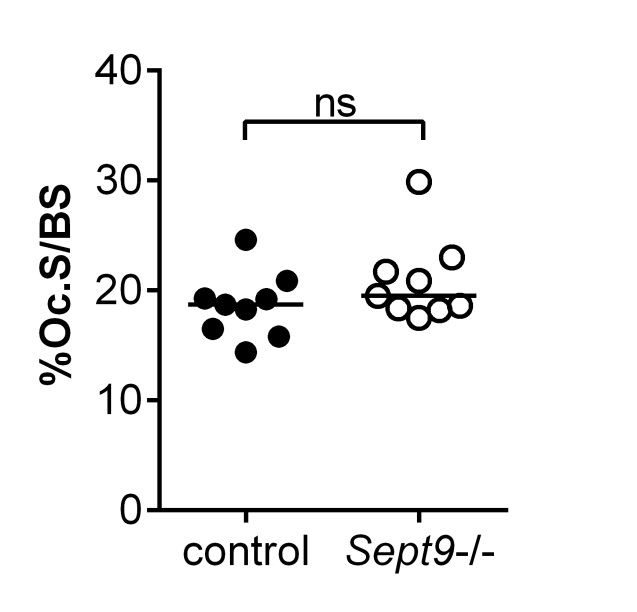


**Osteoclastogenesis appears unaffected by *Sept9* deletion *in vivo*.**

Sequential 3.5-µm-thick vertical paraffin sections were cut of L1-2 vertebrae and histochemically stained for TRAcP activity. A single section from the centre of each L1-2 vertebrae was scanned and used to estimate osteoclast surface per bone surface (Oc.S/BS) using a Mertz grid. Both endo-cortical and trabecular surfaces were counted and pooled obtaining a mean of 874 intercepts per tissue section (range: 292 to 1549 intercepts). Horizontal lines represent the median. Statistics: Mann-Whitney test; ns, not significant, p=0.27.
